# Cognitive impairment in schizophrenia: aetiology, pathophysiology, and treatment

**DOI:** 10.1038/s41380-023-01949-9

**Published:** 2023-01-23

**Authors:** Robert A. McCutcheon, Richard S. E. Keefe, Philip K. McGuire

**Affiliations:** 1 Department of Psychiatry, University of Oxford, Oxford, UK; 2 Department of Psychosis Studies, Institute of Psychiatry, Psychology & Neuroscience, London, UK; 3 Oxford health NHS Foundation Trust, Oxford health NHS Foundation Trust, Oxford, UK; 4 Departments of Psychiatry and Behavioral Sciences, Duke University Medical Center, Durham, NC USA; 5 NIHR Oxford Health Biomedical Research Centre, Oxford, UK

**Keywords:** Neuroscience, Schizophrenia

## Abstract

Cognitive deficits are a core feature of schizophrenia, account for much of the impaired functioning associated with the disorder and are not responsive to existing treatments. In this review, we first describe the clinical presentation and natural history of these deficits. We then consider aetiological factors, highlighting how a range of similar genetic and environmental factors are associated with both cognitive function and schizophrenia. We then review the pathophysiological mechanisms thought to underlie cognitive symptoms, including the role of dopamine, cholinergic signalling and the balance between GABAergic interneurons and glutamatergic pyramidal cells. Finally, we review the clinical management of cognitive impairments and candidate novel treatments.

## Introduction

**Fig. 1:**
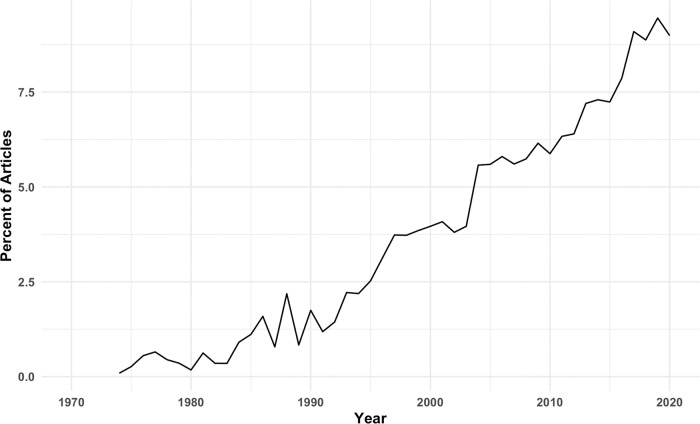
Increasing interest in cognitive impairment in schizophrenia. The graph illustrates the proportion of PubMed articles on schizophrenia that include ‘cognitive impairment’ in the title.

Individuals with schizophrenia show a substantial impairment in overall cognitive performance, which, on average, is around two standard deviations below that in healthy controls [1]. Moreover, this deficit contributes to poor clinical outcomes such as unemployment and an inability to live independently [2]. While cognitive function in schizophrenia is an area of increasing research interest (Fig. 1) [3], this has yet to translate into the development of novel treatments for cognitive problems. All currently approved pharmacological treatments for schizophrenia exert their effects via antagonism of the dopamine D2 receptor [4, 5]. This mechanism of action is efficacious for symptoms that are thought to be driven by excessive striatal dopamine signalling, such as hallucinations and delusions. However, antipsychotic medications have little impact on cognitive impairments in schizophrenia, perhaps because the latter are related to different pathophysiological processes [5]. In the current paper, we outline the clinical nature of cognitive impairment in schizophrenia and consider potential aetiological factors. We then discuss pathophysiology, before concluding with an examination of current and potential future treatment options.

## The nature of cognitive impairments in schizophrenia

### Cognitive deficits appear to be distinct from positive and negative symptoms

Factor analyses of the Positive and Negative Syndrome Scale (PANSS) indicate that a five-factor model (positive, negative, disorganised, excited, and depressed) captures the symptom structure of schizophrenia better than the original a priori grouping of positive, negative, and general symptoms [6, 7]. Of these five factors, the disorganisation factor (which includes difficulty in abstract thinking, poor attention, disorientation, stereotyped thinking and conceptual disorganisation) shows the strongest association with cognitive test scores, but still only accounts for a small proportion of the variance [8, 9]. Network analyses have identified broadly similar symptom groupings, and again find that cognitive scores are distinct from positive and negative symptoms, although linked to disorganisation [10, 11]. Deficits in social cognition are also apparent in individuals with schizophrenia, and these too are separable both from the five PANSS factors and other cognitive domains [12, 13]. Social cognition also has a major impact on functioning [13], and may have distinct pathophysiological underpinnings. This important aspect of cognition in schizophrenia has been reviewed in detail elsewhere [14, 15], and is not within the scope of the present review.

### Are impairments global or domain-specific?

For a given cognitive domain, patients with schizophrenia perform around one standard deviation below the level of controls. However, the psychometric properties of composite scores are such that they are typically more extreme than their constituent parts [16]. Thus, patients show an impairment of around 1.5 standard deviations on overall composite scores relative to controls [1, 17–22]. An unresolved issue is whether there are distinct domains of cognitive impairment in schizophrenia, or whether the deficits are better summarised as global. This issue maps to a parallel debate over whether the pathophysiology of schizophrenia involves specific loci of brain dysfunction or a systems-level disruption.

Factor analyses of the Measurement and Treatment to Improve Cognition in Schizophrenia test battery identified seven cognitive domains: Processing speed, Attention, Working memory, Verbal learning and memory, Visual learning and memory, Reasoning, and Social cognition [23]. Further dimensionality reduction suggests that these seven domains can be reduced to parent domains of Processing speed, Attention/Working memory and learning [24].

Some reports indicate that processing speed is the domain most affected in schizophrenia, and that processing speed deficits are the strongest predictor of general cognitive performance [24–27]. However, processing speed is particularly affected by antipsychotic medications [28], and in medication-naïve cohorts, the magnitude of this impairment is no greater than that for verbal or working memory [19]. Because processing speed is a component of many cognitive functions, deficits in this domain may reflect impairments in other higher-order functions. For example, patients with schizophrenia may approach processing speed tests, such as symbol-coding, less strategically than controls [29]. Equally, impairments in even lower-level domains such as reaction time could contribute to deficits in higher-order domains [23]. Given the existence of severe impairments of low-level processes, and the presence of deficits across many domains, it could be argued that cognitive impairments in schizophrenia are best conceptualised as a generalised deficit. However, it is unclear whether the limitations of existing cognitive tests are such that they cannot isolate specific cognitive processes, precluding the capture of more specific impairments [30, 31]. Tests that can examine more specific cognitive and perceptual processes may be more sensitive to the non-generalised components of cognitive impairments in schizophrenia.

### Variation in cognitive impairments between and within individuals

**Fig. 2:**
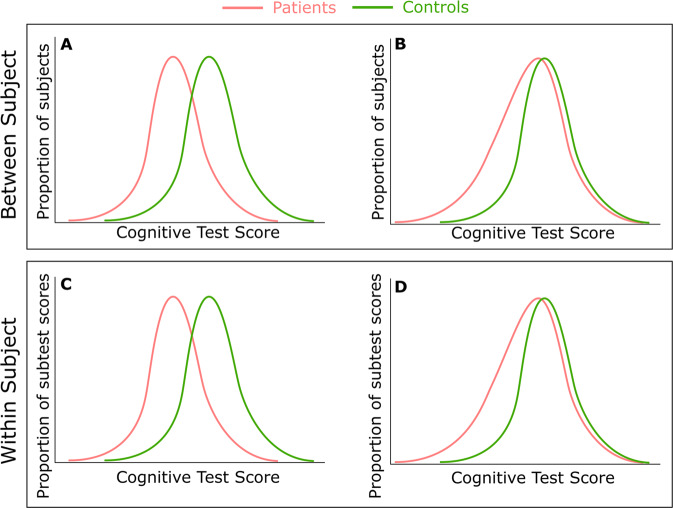
Variability of cognitive function in schizophrenia. Examining the population distribution of cognitive ability can help to determine whether impairments reflect a generalised deficit or are greater in magnitude in some patients than others. Recent data indicate that the distribution is more like that shown in (**B**) than (**A**), with more variability in the patient than the control sample. This suggests in psychosis, rather than there being a constant effect on cognition, in some individuals, there is a large impairment, but in others relatively little. It is also valuable to look at intra-individual variability, unlike (**A**) and (**B**) which represent data from many individuals, (**C**) and (**D**) represent data from a single individual. From this perspective variability again appears greater in schizophrenia and those at risk with the data resembling the distribution in (**D**) more than that in (**C**).

Cognitive functioning appears to vary significantly between individual patients with schizophrenia [32]. Cluster analyses of raw test scores point to a heterogeneity of cognitive function similar to that seen in samples of healthy controls. While these findings provide evidence for clusters of cognitive performance similar to those observed in the general population, they do not demonstrate that the population of patients with schizophrenia comprises clusters of individuals with distinct patterns of cognitive impairment. Thus, it is still unclear whether there are different forms of cognitive impairment across patients, or a generic impairment which is differentially expressed due to variations in premorbid cognitive ability. Analysis of the variability of cognitive functioning between people at clinical high risk (CHR) for psychosis indicates that there is significantly greater variability among these individuals than among controls across a wide range of cognitive domains. This result suggests that there is a heterogeneity in the magnitude of the impairment across individuals, rather than a uniform deficit (Fig. 2) [33].

Variability within individuals has also been examined, and it appears that both over time and across cognitive domains individuals with schizophrenia and those at risk of the disorder display greater intra-individual variability than control subjects [34, 35]. These findings suggest different individuals may show impairments in different domains. However, precisely delineating this is more challenging, and may require prospective studies that begin even earlier than the clinical high-risk stage, before the first expression of symptoms.

### Time course of cognitive deficits in schizophrenia

**Fig. 3:**
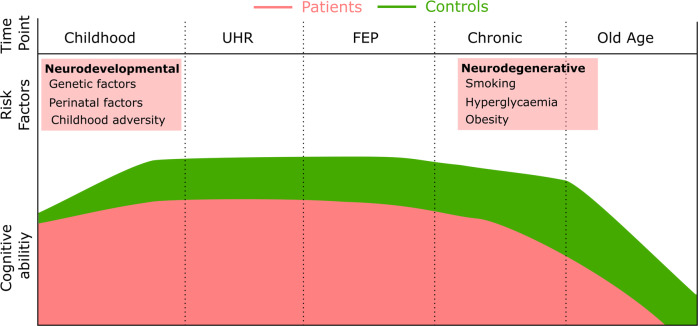
Aetiological factors and time course of cognitive impairments in schizophrenia. Cognitive deficits in individuals who develop schizophrenia are apparent in childhood and do not appear to increase markedly in the initial phase of the illness. While CHR individuals as a group score higher than FEP individuals, longitudinal studies do not provide clear evidence for a decline over the period of transition to psychosis. The decline in cognitive function that occurs in later life in healthy individuals is evident at an earlier age in individuals with schizophrenia, potentially related to neurovascular factors.

In people who later develop schizophrenia, relatively global impairments in cognitive function are detectable in childhood [36], and there is an increase in the severity of nonverbal deficits during adolescence due to the slower development of these abilities (Fig. 3) [37, 38]. This alteration in developmental trajectory over adolescence is a stronger predictor of the subsequent onset of schizophrenia than a cross-sectional impairment in cognitive performance at the age of 18 [39].

Much of the impairment in cognitive functioning that is evident in adults with schizophrenia is thus established before the first expression of symptoms [40] or contact with mental health services. This raises the possibility that even in patients in whom cognitive deficits are not clinically obvious, a degree of deterioration relative to the premorbid state has already occurred. This is supported by the finding that people with schizophrenia, when matched to controls with the same current IQ, have higher scores on cognitive tests that are sensitive to premorbid IQ, but lower scores on working memory (which is not) [32, 41].

The first episode of schizophrenia is often preceded by a CHR state in which cognitive deficits are also observed [42]. Individuals at CHR may or may not progress to develop schizophrenia [43], and cognitive deficits are most severe in the subgroup of the CHR population that subsequently develops the disorder [42]. Whether the transition to frank psychosis in these individuals is associated with a further decline in cognitive function remains an important question. A progression of cognitive impairments (as well as psychotic symptoms) would suggest that neurobiological changes occurring over this period underlie the changes in cognitive function. Alternatively, an absence of progression would indicate that the neurobiological factors critical to cognition are pre-existing and neurodevelopmental. At present, there is no evidence of a decline in raw cognitive performance following transition, although this could reflect practice effects [44]. However, there is some evidence for a relative decline: a recent meta-analysis found that CHR individuals who develop psychosis display a relative worsening in processing speed subsequent to baseline compared to CHR individuals who did not transition [45].

A decline in cognitive function over the course of the illness was originally seen as a defining feature of schizophrenia [46]. Meta-analyses of longitudinal studies, however, have tended to show improvement across multiple cognitive domains. However, many of the studies examined were of limited duration and the observed improvements are likely artefacts of practice effects, with less improvement observed in patients than in controls [47–49]. Long-term longitudinal studies of over 10 years duration are rare, and have shown both improvement [50] and decline [51, 52] in cognitive function. It is difficult in these studies, either due to lack of controls, relatively small sample sizes, or poor matching to controls, to disambiguate how much observed changes reflect normal cognitive trajectories, versus how much is due to a changing magnitude of impairment. Large-scale cross-sectional studies have suggested that while cognitive functioning decline with age this follows normal trajectories with no increase in the severity of impairment [53]. There is some evidence for a more rapid decline in cognitive function over the age of 65 in institutionalised individuals with schizophrenia than controls [54]. The basis of this decline is unclear, but could partly be secondary to poor physical health in patients with schizophrenia, which becomes increasingly evident in later life (Fig. 3). Both the lower starting point and this subsequent decline likely contribute to the increased incidence and earlier onset of dementia in individuals with schizophrenia [55].

### Are cognitive deficits in schizophrenia distinct from those in other psychiatric disorders?

During the development of the DSM-5, the addition of cognitive impairment as a new diagnostic criterion for schizophrenia was considered. However, this was not implemented, because cognitive deficits in schizophrenia were regarded as not sufficiently distinct from those in other conditions (such as bipolar disorder) to be of diagnostic value [56–59]. Nevertheless, the pattern of deficits across different psychiatric disorders is not the same. For example, cognitive impairments in schizophrenia are more severe than in bipolar disorder and depression, and are clearly present before the expression of symptoms, which is not the case for bipolar disorder or depression [60, 61] Ironically, subjective cognitive impairment is one of the DSM criteria for the latter disorders. In schizophrenia, however, the use of a subjective criterion is complicated by the lack of insight associated with the disorder, although subjective cognitive difficulties can be used as diagnostic criteria for the CHR state [62].

### Lived experience of cognitive impairment in schizophrenia

Some individuals with schizophrenia complain of subjective cognitive impairments, and these appear to be even more common among people at clinically high risk for the disorder [63]. In addition to their potential pathophysiological and diagnostic significance, these self-reported symptoms are important because they are associated with distress and a reduction in quality of life [64]. At the same time, it is clear that a significant proportion of individuals with schizophrenia have severe cognitive impairments but do not report subjective deficits [65].

Among patients with schizophrenia there is only a weak correlation between subjective reports of cognitive impairment and objective measures of cognitive performance, and the reporting of subjective impairments appears to be higher in patients with comorbid depression [66, 67]. Although the correlation between subjective reports and objective measures is stronger when the analysis is restricted to the 50% of patients who report the greatest subjective impairment, it is still only modest [65]. Similarly, there is minimal correlation between objective and subjective measures of cognition in individuals at clinically high risk of psychosis [63, 68]. The absence of a strong correlation across the psychosis spectrum is in keeping with findings that individuals at high risk of psychosis report a greater severity of subjective impairment than people with schizophrenia, despite having a lesser degree of impairment on objective testing [63, 69]. A lack of insight and higher levels of disorganised symptoms appear to be key factors that contribute to a poor ability to self-assess cognitive abilities [70, 71], and individuals with high scores on self-certainty measures (e.g., endorsing statements such as ‘my interpretations of my experiences are definitely right’) tend to be those with greater cognitive problems [72]. This has relevance for the delivery of treatments for cognition, as individuals with subjective impairments appear to be more willing to engage in therapy, whereas those with more severe objective impairments may not see the need [73].

Despite the importance of cognitive deficits in schizophrenia, the formal assessment of cognitive function is rarely part of the routine clinical care of people with schizophrenia [74]. With limited time and the absence of formal training, it is difficult to assess accurately [75], and most established instruments require a trained assessor and a lengthy assessment period, which patients often find demanding. Clinicians have an increasingly limited time to see patients, and clinical teams often lack access to a neuropsychologist to conduct cognitive assessments. Moreover, in the absence of effective interventions for cognitive deficits, clinicians may reason that quantifying their severity is unlikely to be of benefit to their patients.

### Functional consequences of cognitive impairments

There is a direct correlation between the level of cognitive performance in schizophrenia and the level of real-world functioning [24, 76]. This relationship is particularly strong when functioning is assessed using performance-based measures such as the UCSD Performance-based Skills Assessment (correlations ranging from *r* = 0.4 to 0.8), as opposed to an interview-based assessment (correlations ranging from 0.1 to 0.3) [24, 76].

A key driver of the substantial health costs associated with schizophrenia is admission to the hospital. Cognitive impairment is linked to reduced adherence to treatment, a greater likelihood of hospital admission, and to longer lengths of hospital stay [77–79]. In addition to health costs, schizophrenia is associated with even greater societal costs, as 80–90% of patients are unemployed, and remain so for most of their adult lives [77, 78, 80]. Cognitive impairments, as well as negative symptoms, are a major factor in this lost productivity [78, 81]. Among people with schizophrenia, a greater severity of cognitive symptoms is associated with lower wages, fewer hours worked in supported employment programs, and fewer benefits gained from employment interventions [82, 83]. Measures of functioning are more strongly correlated with measures of cognition in individuals with schizophrenia than in healthy controls [84], consistent with a non-linear relationship between cognitive performance and function in people with the disorder. The latter raises the possibility that in those with more marked deficits, interventions that result in improvements in cognition could have a disproportionately large benefit on the level of functioning.

## The aetiology of cognitive impairment in schizophrenia

### Genetic factors

Both cognitive ability in healthy individuals and cognitive impairments in schizophrenia appear to be highly heritable. The relatives of people with schizophrenia also show cognitive deficits [85, 86], and twin studies suggest that a significant proportion of the variance in cognition and schizophrenia risk is due to shared genetic factors [87]. Schizophrenia is regarded as a polygenic disorder in which multiple genes of small effect increase risk of illness when certain alleles are present together. The same architecture applies to cognitive abilities in the general population [88, 89].

While attempts have been made to characterise the effects of specific alleles [90], most contribute only a minimal degree of risk (median odds ratio 1.05), and are therefore unlikely to have major pathophysiological relevance in isolation [89]. However, functional consequences of increased genetic risk may be detected by assessing polygenic risk scores. Both twin and genome-wide association studies (GWAS) show a strong negative genetic correlation exists between liability for schizophrenia and cognitive ability, indicating that they share common genetic factors [88, 91–94]. However, this negative genetic correlation is minimal or absent between polygenic risk for bipolar disorder and cognitive function [88, 91, 93, 95]. This is remarkable, given the overlap in genetic risk factors for schizophrenia and bipolar disorder [96]: cognitive function appears to be one phenotypic component that reflects a fundamental difference in their respective genetic architectures. Consistent with this, in healthy cohorts, a high polygenic risk score for schizophrenia is associated with poorer cognition, and low polygenic scores for cognition are associated with an increased risk of schizophrenia [97]. The polygenic risk score for bipolar is also associated with poorer cognition in childhood, primarily as a result of single-nucleotide polymorphisms shared with schizophrenia [98], but bipolar disorder-specific risk alleles are associated with better cognitive performance [99]. Among patients with schizophrenia, cognitive performance is correlated with the polygenic risk scores for IQ and for educational attainment, but not that for schizophrenia [95, 100]. Although this lack of direct correlation suggests that cognitive impairment in schizophrenia is not a consequence of genetic liability for the disorder, cognition may a mediating factor through which genetic risk exerts its effects [101]. As mentioned above, in healthy controls there is an association between higher polygenic risk scores for schizophrenia and lower baseline cognitive performance; however, there is no association with greater cognitive decline, implicating neurodevelopmental rather than neurodegenerative processes [102]. Conclusions drawn from polygenic risk score analyses must be tempered by the fact that these typically account for less than 10% of risk variance, and at present, inferences can only be made in relation to people of European ancestry [103].

Another approach to unpicking GWAS results is to examine associated groups of functionally related genes to determine if genetic pathways are implicated. Counter-intuitively, given the negative correlation at the phenotypic level, these studies have reported a positive correlation between polygenic risk scores for schizophrenia and polygenic risk scores for educational attainment [100]. A recent analysis investigated genes that were positively associated with schizophrenia risk but negatively associated with cognitive ability and looked at how this related to genes associated with both positive and negative educational attainment. For the concordant pathway (i.e., positive educational attainment), gene sets enriched for expression in brain tissue and the CHD8 neurodevelopmental pathway were implicated. When examining the discordant pathway, several synaptic pathways were implicated such as ion channels and synaptic density, and when examining enrichment for drug mechanisms of action, voltage-gated calcium channel genes were over-represented [104].

When considering the genetic underpinning of cognitive abilities, it is important to bear in mind that these do not necessarily imply a direct link. As an example, if individuals of a certain ethnicity have less access to educational opportunities, the alleles associated with this phenotype might show a negative association with cognitive ability. In cases such as this, time will be better spent tracking the environmental mechanism mediating the association than investigating the biological function of the alleles.

### Environmental factors

Although the heritability of schizophrenia is substantial at around 80% [105], even identical twins tend to be discordant for the disorder, underlining the importance of environmental influences [106]. Similarly, in the general population, a wide range of environmental factors are associated with cognitive abilities, and these are of greater importance in situations of socioeconomic disadvantage, where they effectively mask genetic heritability [107]. The increased prevalence of negative environmental factors in individuals who develop schizophrenia therefore raises the possibility that similar mechanisms may hold for cognitive impairments in schizophrenia as in the general population, with environmental influences also playing a major role.

In the prenatal period, obstetric complications are a well-established risk factor for schizophrenia [108] and are also associated with lower IQ in both individuals with schizophrenia and in healthy controls [109]. While prenatal infection is associated with risk of schizophrenia, and some prenatal infections are associated with cognitive impairment in the offspring [110], it does not appear that there is a general association with cognitive abilities when evidence of infection is broadly defined [111]. For some infections such as influenza, the deleterious cognitive effects may be more pronounced in individuals who subsequently develop schizophrenia, suggesting that other aetiological factors play a role, or, alternatively, that individuals with schizophrenia represent a group in which a more serious infection is more likely to have occurred [112].

Growing up in an urban environment is linked to a raised incidence of schizophrenia, although the direction of causality remains unclear [113–115]. Some of this association may be mediated by higher levels of socioeconomic deprivation. In the general population, socioeconomic deprivation is associated with poorer educational attainment [116], potentially because living in affluent neighbourhoods is associated with more pro-cognitive exposures [117]. However, there appear to be additional factors linking cognition and urbanicity. In children born preterm, living in an urban environment is associated with lower cognitive development scores, even after controlling for socioeconomic factors [118]. In the general population, growing up in an urban environment is associated with poorer spatial navigation abilities [119], and air pollution is associated with both poorer cognitive function and with an increased risk of developing schizophrenia [120–122]. Exposure to childhood trauma is associated both with an increased risk of developing schizophrenia, and also with lower performance on cognitive batteries in childhood and adolescence [114, 123, 124].

Acute cannabis use is associated with a clear cognitive impairment [125], and regular users also appear to perform worse on cognitive testing [126]. However, among people with schizophrenia, some studies have reported that cannabis users show better cognitive performance than patients who are non-users [127, 128]. This finding appears counter-intuitive, as cannabis use in healthy volunteers and other studies in schizophrenia has been associated with impairments in cognitive function [125, 129, 130]. The association with better performance in schizophrenia may depend on the pattern of cannabis use, as it is mainly evident in infrequent users rather than in regular or dependent users (Chester et al., in submission). The basis of the association is unclear. One possibility is that occasional cannabis use is a proxy for patients in whom cognition is relatively less impaired: the ability to source illicit cannabis requires motivation, and organisational and social skills. Another is that individuals who develop schizophrenia in the context of cannabis use have a relatively less genetic predisposition and less impairment of cognitive function. However, among people with schizophrenia who use cannabis, those with a family history of schizophrenia have better cognitive performance than those who do not. This has been attributed to cannabidiol (CBD) in cannabis exerting a neuroprotective effect [131]. However, most currently available illicit cannabis contains high levels of THC but minimal amounts of CBD [132].

Ethnic minority status is one of the strongest risk factors for the development of schizophrenia, and ethnically minoritized individuals also tend to have poorer educational attainment [114, 133]. When examining specific ethnic groups, there are examples of correspondence between these outcomes. In England, individuals with Caribbean ancestry show both the greatest increased risk of developing a psychotic disorder and also the poorest educational attainment [114, 133]. Similarly, in individuals of South Asian descent, individuals with Pakistani heritage show both poorer educational attainment and an increased risk of schizophrenia that is not seen in those of Indian heritage [133–135]. Among ethnically minoritized individuals, the effect of ethnicity on schizophrenia risk is even greater if they are also a minority in their local neighbourhood [136]. Similarly, an increase in median neighbourhood income is linked to better cognitive test results in black children, but only if they live in an area that has a high proportion of black individuals [137]. Greater exposure to socioeconomic deprivation, childhood trauma, and racism both structural and interpersonal all have the potential to play a role in explaining these associations, and it remains to be determined to what extent the pathways to increased schizophrenia risk and poorer performance on indices of cognitive ability are parallel or intertwined [138].

Individuals with schizophrenia show a further decline in cognitive ability in later life. The latter may reflect an increased prevalence of smoking [139], obesity [140], and hyperglycaemia [141], which can have an adverse effect on cerebrovascular function. Hypertension, diabetes, and metabolic syndrome are all associated with significantly worse cognitive functioning in individuals with schizophrenia [142]. Additional factors include the lack of social and vocational stimulation that can be associated with the disorder [143].

In summary, impaired cognitive function in people with schizophrenia is related to their genetic loading, an increased exposure to environmental factors that are associated with reduced cognitive performance (Fig. 3), and with poor physical health in later life.

## The pathophysiology of cognitive impairment in schizophrenia

**Fig. 4:**
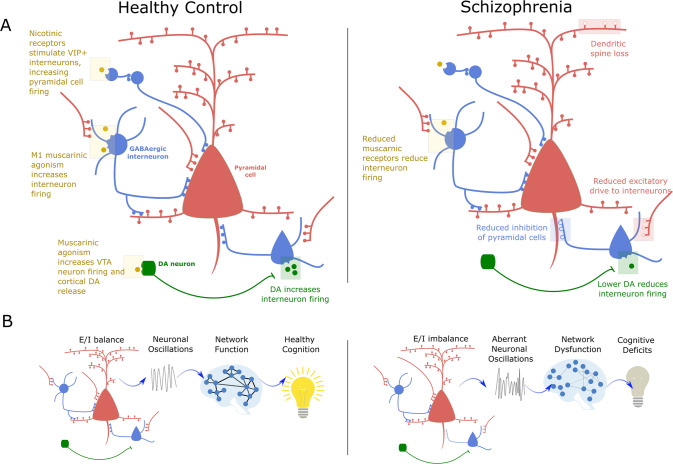
E/I balance as a common pathway to cognitive dysfunction in schizophrenia. **A** Muscarinic, dopaminergic, glutamatergic, and GABAergic influences on E/I balance in healthy controls and mechanisms of disruption in schizophrenia. **B** E/I balance and cognition. In healthy individuals, interactions between excitatory pyramidal cells and inhibitory interneurons generate gamma oscillations, which are associated with functional brain networks observable using fMRI, with all levels showing links to healthy cognitive function. In individuals with schizophrenia, disruptions to muscarinic and dopaminergic signalling, an intrinsic interneuron deficits may underlie a state of cortical disinhibition. This disinhibition would account for the aberrant gamma activity and functional networks observed in the disorder and contribute to cognitive impairments.

A wide range of neurotransmitters and brain circuits are implicated in schizophrenia. Many of these appear to converge on a common pathway fundamental to cognitive functioning, namely the balanced interactions between excitatory and inhibitory (E/I) neurons of cortical microcircuits (Fig. 4). Excitation allows neurons to fire in response to stimuli, while inhibition tunes their responses, allowing for precise neural representations. The balance between the two is crucial for the neural computations underlying cognition. We now discuss the role of dopaminergic, cholinergic, glutamatergic, and GABAergic systems. We discuss the evidence for aberrant function in schizophrenia, the role in healthy cognitive function, and their part in modulating E/I balance.

### Dopamine

Dopamine occupies a central role in the pathophysiology of schizophrenia [144–146]. In preclinical models, increased dopamine signalling is associated with schizophrenia-like behaviours [147]. In patients, post-mortem studies show dopamine receptor upregulation [148], and the potency of antipsychotic medications is tightly linked to their affinity for the D2 receptor [4, 149]. Neuroimaging studies indicate that schizophrenia is associated with presynaptic hyperdopaminergia in the dorsal striatum [150], and experimental stimulation of striatal dopaminergic transmission (e.g., following administration of amphetamine) can induce and exacerbate psychosis [151, 152].

Although dopamine dysfunction has mainly been linked to positive psychotic symptoms, striatal dopamine signalling normally has widespread effects on cortical function [153–156], and its dysregulation can also cause cognitive symptoms [157]. Measures of striatal dopamine signalling appear to index ‘state’ aspects of psychosis, increasing during acute psychosis and demonstrating an association with the severity of positive psychotic symptoms, positioning striatal dopamine as ‘the wind of the psychotic fire’ [158–160]. Cognitive symptoms, however, follow a much more constant trajectory than positive symptoms, and as such, it is less likely that they are directly tied to neurochemical measures that fluctuate over the illness course [161, 162].

In vivo measurement of cortical dopamine signalling is challenging given the relative sparsity of the receptors. However, data from recent studies are consistent with the hypothesis that schizophrenia is associated with a hypodopaminergic state in the cortex [163–165]. Moreover, cortical hypodopaminergia may be linked to striatal hyperdopaminergia in schizophrenia: preclinical work has demonstrated that depletion of cortical dopamine increases striatal dopamine levels [166, 167], and there is preliminary in vivo evidence that the same relationship applies in people with schizophrenia [165]. The lack of effect of dopamine antagonists on cognitive symptoms may reflect the fact that correcting the dysregulated striatal signalling in schizophrenia requires not only the blocking of aberrant signals, but also the restoration of the signal-to-noise ratio of adaptive signals [168].

Cortical dopamine signalling plays a role in normal attention, working memory, and executive function, so impaired dopamine function in schizophrenia may therefore affect these processes [169–171]. Dopamine promotes the persistent cortical activity required for the maintenance of working memory, while inhibiting unrelated signalling so that relevant information is precisely represented [172]. Studies in people with Parkinson’s disease, and in non-human primates with experimental lesions of prefrontal dopamine function indicate that impaired dopamine signalling leads to working memory and executive functioning deficits [173, 174]. Conversely, The effectiveness of pro-dopaminergic compounds in the treatment of ADHD suggests that augmentation of cortical dopamine signalling has the potential to exert cognitive benefits [175]. Recently the dopaminergic partial agonist cariprazine has demonstrated some advantages on cognitive subscales of the PANSS when compared to antagonists, but more in-depth testing of its cognitive effects is required [176].

However, despite some promising signals in experimental medicine studies [177], pro-dopaminergic treatments have not been approved as treatments for the cognitive deficits of schizophrenia. This may reflect the complexity of the system involved. The effects of dopamine in the cortex are distinct to those in the striatum, and only a fraction of cortical pyramidal neurons express dopamine receptors, with cortical interneurons showing proportionally greater expression [178]. For both pyramidal cells and interneurons, dopamine’s presynaptic effects predominantly reduce neurotransmitter release. Postsynaptically, dopamine enhances the excitability of deep-layer pyramidal neurons, and increases the frequency of interneuron spiking. The net effect of these various mechanisms is that cortical dopamine depresses pyramidal cell firing via its action on interneurons. A link with E/I balance is therefore apparent, in that reduced cortical dopamine signalling is associated with a reduced net inhibitory input, termed disinhibition.

### Acetylcholine

Acetylcholine plays a central role in attention and memory. Treatment with anticholinergic drugs can lead to cognitive impairments in these domains as a side effect, and the majority of medications used for treating symptoms of Alzheimer’s disease operate by promoting cholinergic signalling [179, 180]. Central cholinergic innervation is split between two primary networks, the pedunculopontine cholinergic complex which projects to the midbrain, and the forebrain complex which projects to the cortex. The effects of acetylcholine are mediated by two receptor types—ionotropic nicotinic receptors and G protein-coupled muscarinic receptors.

A link between the nicotinic system and schizophrenia is supported by the strikingly high prevalence of tobacco dependence, with around 65% of patients being smokers [181]. This high prevalence has persisted despite clinical initiatives to reduce smoking in patients with schizophrenia, which is a key factor in the 15–20-year reduction in life expectancy associated with the disorder [182]. Most patients are aware of this health risk, but report that they continue to smoke because it improves their concentration and reduces anxiety [183]. This is in keeping with evidence that the acute administration of nicotine ameliorates sensory gating abnormalities and enhances cognitive performance in schizophrenia [184, 185]. However, this enhancement is not seen with chronic use [186], and in the longer term, smoking in schizophrenia is associated with poorer cognitive performance and increases the risk of late-life cognitive decline [187]. Furthermore, smoking cessation appears to improve cognition in schizophrenia [187]. The immediate benefits of tobacco smoking may arise from initial agonism at cholinergic receptors, with the deleterious effects resulting from receptor desensitisation due to chronic exposure. In addition, in people with schizophrenia, the repeated stimulation of nicotinic receptors by smoking leads to less of a reduction in nicotinic receptor expression than in control smokers [188]. Despite this strong association between cholinergic signalling and cognition in schizophrenia, agents that affect nicotine receptor function such as varenicline, or that target cholinergic neurotransmission more broadly, like acetylcholinesterase inhibitors have had disappointing results when used to treat the cognitive deficits of schizophrenia [189, 190].

A separate body of evidence suggests that schizophrenia is associated with aberrant functioning of the muscarinic system. Acute administration of muscarinic antagonists can exacerbate both cognitive deficits and positive symptoms in individuals with schizophrenia, and can induce cognitive deficits and positive symptoms in controls [191]. The G protein-coupled muscarinic receptors either have predominantly excitatory (M1, M3 and M5 subtypes) or inhibitory (M2 and M4) effects. Post-mortem studies in schizophrenia show significant reductions of both M1 and M4 receptors in hippocampus and cortex [191–193]. Similarly, PET studies have found reduced muscarinic receptor density in schizophrenia, and have linked this to the presence of cognitive symptoms [194–196]. M1 receptors play an important role in learning and memory [197], and M1 agonism may improve both positive symptoms and cognitive performance in people with schizophrenia [198–200].

Both nicotinic and muscarinic receptor systems affect GABAergic and glutamatergic signalling and can thereby contribute to the maintenance of E/I balance [197]. Nicotinic receptors are sparsely distributed on pyramidal cells but are more frequently expressed on interneurons, particularly those expressing vasoactive intestinal peptide (VIP) [201–203]. VIP interneurons predominantly inhibit other interneurons, and the net effect of nicotinic stimulation upon E/I balance is therefore to increase pyramidal cell firing. This effect may be beneficial if there is reduced pyramidal signalling, but is unlikely to be helpful if there is a state of cortical disinhibition, as may occur in schizophrenia. In contrast to nicotinic receptors, M1 receptors are widely expressed on parvalbumin-positive interneurons and their activation enhances GABA release, playing an important role in learning and memory [197]. M1 receptors are also strongly expressed on pyramidal cells, suggesting that the net effect of M1 agonism might be to increase cortical excitability. Recent work, however, demonstrates that both M1 agonism and antagonism have a net inhibitory effect on cortical pyramidal neurons. The overall effect of modulating activity at M1 receptors is likely to depend on the degree of endogenous tone: at typical levels, this may already be saturated, such that effects are primarily inhibitory via increased activation of interneurons, or a direct effect on pyramidal cells via other pathways [204]. These effects on E/I balance are evident in the modulation of gamma oscillations by muscarinic compounds [205]. Muscarinic agonism is therefore likely to produce cognitive benefits when there is a state of cortical disinhibition, which the evidence presented below suggests occurs in schizophrenia.

In addition to the links with excitation and inhibition, cholinergic signalling also impacts on the dopamine system. At low agonist concentrations, stimulation of nicotinic receptors on GABAergic projections to mesostriatal dopamine neurons inhibits dopamine release, while at higher concentrations this mechanism becomes saturated and stimulation of receptors on glutamatergic projection neurons increases dopamine neuron firing [206]. Stimulation of M4 muscarinic receptors on striatal cholinergic interneurons can reduce acetylcholine release and thereby minimise cholinergic excitation of dopamine neurons projecting to the striatum. M4 agonism can also reduce striatal dopamine release by inhibiting the activity of spiny projection neurons to the midbrain [207, 208]. In contrast, and of particular interest given the reduced cortical dopamine signalling observed in schizophrenia, M1 agonism appears to increase cortical dopamine release [209–211].

### E/I balance

GWAS have demonstrated that SNPs associated with schizophrenia are concentrated in genes expressed in E/I neurons [89]. Post-mortem studies, in line with a model of cortical disinhibition, have found reductions in the enzymes GAD65 and GAD67, which are required for GABA synthesis, as well as reduced excitatory input to inhibitory neurons, and reduced interneuron numbers [212, 213]. A wide range of animal models that display cognitive deficits analogous to those observed in schizophrenia, demonstrate cortical disinhibition as a common final condition underlying pathological behaviour [214, 215].

E/I balance in humans can be assessed using neuroimaging. Magnetic resonance spectroscopy (MRS) can measure glutamate and GABA concentrations at rest, and functional MRS (fMRS) can examine how these dynamically change in the face of a cognitive challenge [216–218]. Studies in individuals with schizophrenia have demonstrated increased Glx (glutamate and glutamine signal combined) [219] and reduced GABA [220], consistent with underlying cortical disinhibition. However, it is not possible to disambiguate intracellular and intrasynaptic signals in MRS data, and there is heterogeneity in the direction of reported findings, which may reflect differences in acquisition methods and medication exposure as much as pathophysiological heterogeneity [219, 221–223]. Nevertheless, an increase in patients who do not benefit from standard treatment with dopamine antagonists appears to be a relatively robust finding [220, 224, 225]. fMRS shows that cognitive challenges induce an increase in the ratio of glutamate:GABA concentrations, and there is preliminary evidence that this increase is reduced in schizophrenia [226, 227]. PET ligands can provide a greater level of molecular specificity than MRS. There have been fewer studies, but these have suggested a reduction in both GABA and NMDA receptors [228–230]. In addition, reduced availability of the metabotropic glutamate receptors type 5 has been linked to cognitive symptoms in schizophrenia [231].

E/I can also be investigated using electroencephalography (EEG). Interactions between pyramidal cells and interneurons generate neuronal oscillations in the gamma (γ) frequency range (Fig. 4) [232, 233]. In controls, gamma oscillations are associated with working memory, and memory tasks elicit an increase in pyramidal neuron firing which is manifest as an increase in gamma power [234, 235]. Cortical disinhibition is associated with increased resting gamma power, and a reduced ability to mount the normal increase in gamma power in the face of cognitive demands [215, 233, 236–239]. Several studies suggest that schizophrenia is associated with both increased resting gamma power and reduced task-induced increases in power [240–247], consistent with a state of cortical disinhibition. Furthermore, reduced mismatch negativity (an EEG-detectable response to surprising stimuli) is a marker of cortical disinhibition [248, 249], and is observed in psychosis where it is associated with cognitive symptoms [250–254].

Gamma oscillations are associated with the emergence of whole-brain functional networks observable using fMRI (Fig. 4) [255–257]. These fMRI-derived networks are associated with cognition in healthy individuals, and with the cognitive symptoms of schizophrenia [258, 259]. Computational models have demonstrated how a global disruption of cortical disinhibition may be manifest through regionally varying patterns of aberrant resting state fMRI activity that mirror those observed in schizophrenia [260–263]. These models indicate that despite the ubiquitous nature of glutamatergic and GABAergic signalling, even a globally present microscale deficit may result in non-uniform effects on macroscale neural dynamics across the cortex, as the wiring patterns of the brain are non-random [261]. Regions that are situated centrally in terms of network dynamics may be where functional effects become concentrated and therefore appear to reflect focal alterations in neuroimaging studies, despite having a spatially distributed molecular basis [264]. Nevertheless, however, focal effects at a molecular level are also possible. For example, because parvalbumin interneurons have extremely high energy requirements, oxidative and metabolic stressors can have a disproportionate impact on these neurons, and their effects may be greatest in regions where they are present at high densities [265].

Thus, while the model of E/I imbalance discussed aims to explain systems-level phenomena, it is not inconsistent with findings that certain brain regions, such as the hippocampus, appear to play a central role in both cognitive function and schizophrenia [266]. Similarly, the functional alterations described above are likely to be interlinked with other well-established aspects of schizophrenia pathophysiology. Aberrations of neurotransmitter function will be coupled, both as cause and consequence, to structural alterations in terms of both anatomical connectivity and grey matter loss. The relevance of structural changes to cognition is seen in recent machine learning studies identifying a pattern of grey matter loss linked to subgroups of patients with cognitive deficits [267–269].

Finally, at the level of behaviour, computational models predict that cortical disinhibition will be associated with a reduced precision of neural representations, and therefore precision-related deficits on working memory tasks [260]. The pattern of working memory deficits observed in both psychosis and pharmacologically-induced disinhibition are in keeping with these predictions [260, 263]. At a higher level, a ‘cognitive map’ refers to the concept that accumulated knowledge and experiences are linked in an organised structure that allows for subsequent novel inferences. Disruption to the architecture of cognitive maps schizophrenia may potentially provide a framework to account for problems in executive functioning and general reasoning abilities [270]. A key consideration in terms of the inferential reasoning supported by a cognitive map is how easily separate memories can be linked: too high a barrier and no connections can be made; too low and entirely unrelated memories may be inappropriately linked. Impairment in this process of memory linkage has recently been demonstrated in schizophrenia [271]. As inhibitory signalling is crucial for appropriate memory separation [272], disinhibition could promote the aberrant associations and working memory deficits that are evident in people with schizophrenia [270].

There is thus multimodal evidence that E/I balance is disrupted in schizophrenia and is associated with cognitive deficits. While we have highlighted findings consistent with a pattern of cortical disinhibition, the nature of the primary deficit has not been firmly established. One hypothesis is that the loss, and/or aberrant function of parvalbumin-positive interneurons (e.g., secondary to NMDA receptor hypofunction, or reduced dopaminergic stimulation) reflects a direct pathophysiological process, with the implication that augmenting the activity of this cell type could ameliorate cognitive deficits. This would be consistent with the ability of the NMDA antagonist ketamine to induce acute psychotic symptoms in healthy volunteers [273], and the finding that anti-NMDA autoantibodies may cause acute psychosis [274]. Moreover, animal models of interneuron dysfunction result in the expression of schizophrenia-like phenotypes, and various computational models have linked cortical disinhibition to schizophrenia [215, 260, 261]. However, it is also possible that the reduced activity of interneuron populations reflects an adaptive response to a primary reduction in cortical pyramidal cell activity. According to this model, a reduction in inhibitory interneuron activity would restore pyramidal cell firing, so novel treatments designed to increase interneuron activity would therefore be expected to have a deleterious effect. Supporting this interpretation are post-mortem work suggesting that schizophrenia is associated with a reduction in the density of dendritic spines on pyramidal cells, in vivo findings demonstrating reduced synaptic density in the cortex [275–277], and data from biophysical network models of fMRI and MEG data pointing to a reduction in synaptic gain on pyramidal cells [278]. It is also consistent with the decline in cognitive function seen in schizophrenia during adolescence, a period when the pruning of dendritic spines is at its peak [279]. A key outstanding question is thus whether reduced inhibitory signalling in schizophrenia represents a primary pathology which treatment should aim to ameliorate, or a compensatory mechanism which, conversely, treatment should try to further reduce.

## The treatment of cognitive impairment in schizophrenia

### Existing treatments

All medications currently licensed for the treatment of schizophrenia exert their clinical effects via antagonism of the dopamine D2 receptor, and are described as antipsychotics. In the 1990s, the introduction of the so-called ‘atypical’ antipsychotics was accompanied by trials suggesting that these newer types of antipsychotic medication had beneficial effects on cognitive symptoms, in addition to psychotic symptoms. Subsequent research, however, suggested that these purported advantages may have been artefacts of trial design, and the current literature suggests that all antipsychotics have similarly small effects on cognitive function [280].

Other medications used in the management of schizophrenia may also have effects on cognition. The anticholinergic effects of many psychotropic medications have a clear detrimental effect on cognitive function, and reducing the dose of anticholinergic medications used to ameliorate extrapyramidal side effects of antipsychotics can improve cognitive function in schizophrenia [281, 282]. Affective symptoms like depression are often associated with cognitive symptoms [283, 284], and while the non-selective administration of antidepressants in schizophrenia does not appear to have pro-cognitive effects [285], the benefits on cognitive function from their selective use in the subgroup of patients with schizophrenia that have prominent depressive symptoms have yet to be assessed.

In the longer term, treating the high prevalence of obesity, type II diabetes and cardiovascular illness in patients with schizophrenia has the potential to minimise the cognitive decline associated with these comorbidities. However, while there has recently been great progress in the detection and monitoring of physical health problems in schizophrenia, the impact of interventions targeting these is only beginning to be investigated [286]. Sleep disturbance is also common in schizophrenia [287], and given the well-established links between sleep and cognitive function [288], addressing sleep problems may also lead to cognitive benefits in schizophrenia. However, treating sleep dysfunction is difficult, and the effectiveness of this approach in people with schizophrenia is unclear. Cognitive remediation therapy in schizophrenia has been shown to improve cognitive test scores, although as a significant component of the intervention involves the repeated practice of cognitive tests there is a question as to how much this represents practice effects. However, there do appear to be detectable benefits on level of functioning, although of relatively small effect size [289, 290]. Nevertheless, at present, no psychological interventions have been approved for the treatment of cognitive symptoms in schizophrenia [291]. Psychological treatments for impairments in social cognition have a less established evidence base but also show some potential [292]. For a more in-depth discussion of potential mechanisms of psychological interventions for cognitive deficits see recent reviews [293, 294].

### Novel treatments

Trace amine-associated receptor 1 (TAAR1) agonists can reduce central dopamine signalling by reducing midbrain dopamine neuron firing [295]. TAAR1 agonism has recently been reported to reduce psychotic symptoms in schizophrenia [296]. Although its effects on cognitive symptoms have yet to be examined, in animal models of schizophrenia it has been found to improve cognitive performance and increase prefrontal cortical activity [297]. The precise mechanisms underlying these latter effects are unclear. Targeting presynaptic neuronal activity may have effects on dopamine signalling that are different from the antagonism of post-synaptic D2 receptors, a hypothesis supported by the absence of extrapyramidal side effects associated with TAAR1 agonism [296]. There is also evidence that TAAR1 agonism affects E/I balance [298].

While the augmentation of cholinergic signalling via drugs that inhibit acetylcholinesterase provides some benefit to cognitive function in Alzheimer’s disease, application of this approach in schizophrenia has not been successful [299, 300]. This suggests a more targeted approach to modulating the cholinergic system may be required. The M1/M4 receptor agonist xanomeline appears to improve both positive and cognitive symptoms in randomised controlled trials in schizophrenia, as well as in Alzheimer’s disease [198, 199, 301]. Initial development of xanomeline was paused due to the high incidence of peripheral pro-cholinergic side effects, but combining it with trospium, a peripheral cholinergic antagonist, markedly reduces its side effects without affecting its efficacy [199]. As discussed above, the mechanism of action here likely involves effects on both dopamine signalling and on E/I balance.

CBD has become increasingly recognised as a potentially effective treatment for schizophrenia. The largest trial of adjunctive CBD in schizophrenia to date demonstrated a clear benefit of CBD on positive symptoms. Although this study was not powered to assess effects on cognition, there was a trend for an improvement in the speed of processing [302]. Human imaging studies suggest that the effects of CBD may be mediated by modulating hippocampal and striatal function [303], but CBD acts on a variety of molecular targets, and the precise mechanism underlying its therapeutic effects remains unclear [304]. However, its efficacy in treating seizures, ability to improve cognition in a mouse model of E/I imbalance, and electrophysiological data from rodents suggest that it may modulate E/I balance, and this may involve antagonising the G protein-coupled receptor GPR55 [305–307].

A wide range of compounds targeting the glutamate system directly have been tested as potential treatments for cognitive dysfunction in schizophrenia, but the results have been disappointing [308, 309]. Agonism of metabotropic mGlu2/3 receptors was initially seen as promising and led to large-scale trials, but development ceased when these failed to show an effect on positive or negative symptoms [310, 311], and the effects on cognitive impairments were not directly tested. Both riluzole and memantine, drugs approved for the treatment of motor neuron disease and Alzheimer’s disease respectively, have complex effects on the E/I system and have shown promise as treatments for cognitive symptoms in schizophrenia [312, 313]. Although demonstrating efficacy on cognitive symptoms in meta-analyses, memantine did not show cognitive benefits in a large trial, although this was in an entirely unselected patient population [314]. More recently a glycine transport inhibitor has demonstrated efficacy as an augmentation agent for treating cognitive symptoms [315]. There is also preliminary evidence that luvadaxistat, a D-amino acid oxidase inhibitor that augments glutamatergic signalling by elevating serine levels, may have beneficial effects on cognitive symptoms [316].

## Future directions

There remain several questions as to the nature of cognitive deficits in schizophrenia. Phenotypically similar cognitive impairments are also evident in other psychiatric disorders, mainly differing from those in schizophrenia in terms of their severity. It is unclear whether the cognitive deficits in these conditions have the same pathophysiological basis as those in schizophrenia. If so, then transdiagnostic approaches to developing interventions may be of benefit. Identifying whether variation in cognitive performance in schizophrenia relates to variation in the severity and nature of the illness, as opposed to variation in premorbid function also has relevance for whether precision psychiatry approaches (which aim to stratify patients according to differing underlying pathophysiologies [280]) are likely to be indicated. In the case of the former, it may be possible to match specific treatments to distinct pathophysiological mechanisms, whereas the latter case represents a uniform insult and so individual differences in treatment effects are less likely.

In terms of work with direct clinical relevance, clinical guidelines recommend the assessment of cognition in standard clinical practice [317], and raising clinician awareness of the need to formally assess cognitive function will become increasingly important as novel interventions become available. It is therefore critical that clinicians are provided with the tools to facilitate the measurement of cognitive impairments so that they can assess the effect of treatment. Traditional cognitive batteries are too time consuming for use in routine clinical care, but the introduction of briefer batteries that can be administered without a trained assessor has major potential here.

In clinical trials, there may be an advantage in evaluating candidate treatments in specific patient subpopulations. Recruiting patients early in the illness course may be of benefit, as although most cognitive deficits will already be present, the higher degree of neuronal plasticity at this stage may increase the chances that intervention will have beneficial effects. Studying individuals early in the disorder also reduces the likelihood that outcomes will be confounded by effects of prior drug treatment. Another subgroup that could be targeted is patients with the greatest degree of cognitive impairment. A recent trial of xanomeline-trospium in schizophrenia found that its impact on cognition was restricted to those with a prominent degree of impairment at baseline [199]. However, it could also be argued that patients with relatively severe cognitive deficits are less likely to improve, due to the severity of neurobiological damage or a high burden of risk factors. To date, most trials of interventions designed to improve cognition in schizophrenia have recruited patients without stratifying samples according to the severity of cognitive impairments. Until it is clear whether enriching samples for patients with marked cognitive deficits will increase or decrease the detection of therapeutic effects, it may be sensible to continue with this approach.

Tying the effects of interventions to real-world functional outcomes is a major challenge but is critical if the aim is to produce outcomes that are meaningful to patients. The use of virtual reality tools may be of benefit here [281]. Non-linear relationships have been observed between symptom dimensions and cognition in schizophrenia [318, 319]. If non-linearities are also seen in the relationship between cognition and function, it is possible that cognitive impairments may only have marked effects on a patient’s level of functioning when they exceed a certain severity threshold. Clarifying the nature of this relationship in schizophrenia could be a goal for future research. If multiple medications are found to be effective in clinical trials precision psychiatry approaches will be important to optimise clinical benefits. Finally, trials aimed at determining whether novel treatments produce transdiagnostic improvements, or whether these are diagnosis specific has major relevance for both clinical practice and development of future treatments.

## Conclusion

Over the past three decades, cognitive impairment has emerged as an increasingly important treatment target for schizophrenia. However, the complexity of the neurobiological substrate has made the development of treatments for these deficits particularly challenging. Nevertheless, there are now a number of clinical interventions which have the potential to improve cognitive function in individuals with schizophrenia, and several new treatments with entirely novel mechanisms of action are ready to be rigorously tested. The development of effective treatments for cognitive impairments in schizophrenia would represent an advance in its treatment comparable to the advent of D2 antagonists over 70 years ago.
