# Correction: Cognitive impairment in schizophrenia: aetiology, pathophysiology, and treatment

**DOI:** 10.1038/s41380-023-01984-6

**Published:** 2023-02-02

**Authors:** Robert A. McCutcheon, Richard S. E. Keefe, Philip K. McGuire

**Affiliations:** 1 Department of Psychiatry, University of Oxford, Oxford, UK; 2 Department of Psychosis Studies, Institute of Psychiatry, Psychology & Neuroscience, London, UK; 3 Oxford Health NHS Foundation Trust, Oxford, UK; 4 Departments of Psychiatry and Behavioral Sciences, Duke University Medical Center, Durham, NC USA; 5 NIHR Oxford Health Biomedical Research Centre, Oxford, UK

**Keywords:** Neuroscience, Schizophrenia

Correction to: *Molecular Psychiatry* 10.1038/s41380-023-01949-9, published online 23 January 2023

In this article the affiliation details for Prof McGuire were incorrectly given as

1. Department of Psychiatry, University of Oxford, Oxford, UK. 2 Department of Psychosis Studies, Institute of Psychiatry, Psychology & Neuroscience, London, UK. 3 Oxford Health NHS Foundation Trust, Oxford Health NHS Foundation Trust, Oxford, UK

The correct affiliations are

1. Department of Psychiatry, University of Oxford

2. NIHR Oxford Health Biomedical Research Centre

3. Oxford Health NHS Foundation Trust

The original article has been corrected.

